# ICP-Based Mapping and Localization System for AGV with 2D LiDAR

**DOI:** 10.3390/s25154541

**Published:** 2025-07-22

**Authors:** Felype de L. Silva, Eisenhawer de M. Fernandes, Péricles R. Barros, Levi da C. Pimentel, Felipe C. Pimenta, Antonio G. B. de Lima, João M. P. Q. Delgado

**Affiliations:** 1 Laboratory of Electronic Instrumentation and Control (LIEC), Department of Electrical Engineering (DEE), Federal University of Campina Grande (UFCG), Campina Grande 58429-900, PB, Brazil; prbarros@dee.ufcg.edu.br (P.R.B.); levi.pimentel@dee.ufcg.edu.br (L.d.C.P.); felipe.pimenta@ee.ufcg.edu.br (F.C.P.); 2 Department of Mechanical Engineering, Federal University of Campina Grande (UFCG), Campina Grande 58429-900, PB, Brazil; antonio.gilson@ufcg.edu.br; 3 CONSTRUCT-BPG, Department of Civil and Georesources Engineering, Faculty of Engineering, University of Porto, 4200-465 Porto, Portugal

**Keywords:** localization and mapping, iterative closest point (ICP), light detection and ranging (LiDAR), automated guided vehicle (AGV), point clouds

## Abstract

This work presents the development of a functional real-time SLAM system designed to enhance the perception capabilities of an Automated Guided Vehicle (AGV) using only a 2D LiDAR sensor. The proposal aims to address recurring gaps in the literature, such as the need for low-complexity solutions that are independent of auxiliary sensors and capable of operating on embedded platforms with limited computational resources. The system integrates scan alignment techniques based on the Iterative Closest Point (ICP) algorithm. Experimental validation in a controlled environment indicated better performance using Gauss–Newton optimization and the point-to-plane metric, achieving pose estimation accuracy of 99.42%, 99.6%, and 99.99% in the position (x, y) and orientation (θ) components, respectively. Subsequently, the system was adapted for operation with data from the onboard sensor, integrating a lightweight graphical interface for real-time visualization of scans, estimated pose, and the evolving map. Despite the moderate update rate, the system proved effective for robotic applications, enabling coherent localization and progressive environment mapping. The modular architecture developed allows for future extensions such as trajectory planning and control. The proposed solution provides a robust and adaptable foundation for mobile platforms, with potential applications in industrial automation, academic research, and education in mobile robotics.

## 1. Introduction

Autonomous Mobile Robots (AMRs) represent a significant advancement over Automatic Guided Vehicles (AGVs), as they offer greater autonomous navigation capabilities by combining sensory data, navigation systems, and control algorithms [1]. Unlike AGVs, AMRs do not rely on fixed tracks or physical infrastructure in the environment, being able to plan and adjust their routes in real time, dynamically adapting to environmental changes [2].

To enable such autonomy, robust perception systems are required, capable of performing localization and mapping simultaneously. Simultaneous Localization and Mapping (SLAM) is a widely used technique for this purpose, allowing the construction of a map of unknown environments while continuously estimating the robot’s position [3,4,5]. Among the various existing approaches, LiDAR (Light Detection and Ranging)-based SLAM stands out for offering high-precision and high-frequency measurements.

In the context of LiDAR-based SLAM, the Iterative Closest Point (ICP) algorithm is one of the most relevant for refining localization and aligning point clouds generated by sensors [6]. ICP iteratively seeks to minimize the difference between two sets of points, traditionally using Euclidean distance to find the corresponding pairs, making it essential for applications in which mapping and localization accuracy is critical [7].

The fundamental problem of SLAM consists of estimating the robot’s pose while simultaneously building a map of the environment, using the reobservation of previously extracted features [4,5]. Consolidated approaches such as Hector SLAM, Gmapping, Cartographer, ORB-SLAM, and LOAM employ different sensors (LiDAR, cameras) and graph-based optimization techniques [8,9,10].

Each method presents specific advantages and limitations: LOAM and Cartographer offer high accuracy but require additional sensors (IMU, 3D LiDAR) and high computational capacity [11,12]; Gmapping depends on odometry and is vulnerable to errors on uneven terrain [9]; and ORB-SLAM and Cartographer are computationally intensive [8,13], while Hector SLAM is efficient and independent of odometry but suffers from error accumulation due to the lack of loop closure [14]. Table 1 presents a comparative overview of the main SLAM approaches discussed, highlighting their sensor requirements, computational complexity, and key features relevant to applications in embedded systems.

Given these limitations, ICP-based methods emerge as promising alternatives for embedded SLAM systems using 2D LiDAR, as they offer a good balance between accuracy, computational efficiency, and independence from complementary sensors [8,14].

Several recent studies have proposed advancements in the use of ICP within the SLAM context: GenZ-ICP, proposed by [15], dynamically adjusts the weight between point-to-point and point-to-plane metrics in 3D LiDAR, improving alignment in degenerate environments, although without real-time validation; Ref. [16] incorporated robust outlier rejection techniques into ICP, but the study was validated only on simulated data. In 2D SLAM, Ref. [17] combined ICP optimized with Levenberg–Marquardt (LM) and Weighted Signed Distance Function (WSDF), increasing reconstruction accuracy at the cost of higher computational demand. The researchers in [18] introduced scan-to-map matching via Discrete Fourier Transform, reducing pose errors but showing sensitivity to map quality. Focusing on efficiency, Ref. [6] created the SLAMICP Library, integrating outlier detection to accelerate processing, though dependent on prior maps; Ref. [19] combined Hector SLAM with LM and smoothed Dynamic A*, improving scan matching stability and reducing pose error. More recently, Ref. [20] presented an ICP-based SLAM system aimed at structured indoor environments, such as multi-level warehouses. The system obtained accurate results, but its execution required higher-performance hardware, and no data on computational consumption were presented.

These advancements highlight recent improvements in computational efficiency, noise robustness, and drift reduction in LiDAR-based SLAM. However, challenges persist, such as the high computational demand of optimized methods, reliance previous maps or additional sensors, and sensitivity to errors in environmental representation.

Thus, there is a clear need for a SLAM solution that operates efficiently on embedded platforms with limited computational resources, is independent of auxiliary sensors, is capable of performing incremental mapping and localization with low error accumulation, and features a modular architecture to support real-time visualization and validation.

Given this scenario, this work presents the development of an incremental real-time mapping and localization system aimed at enhancing the perception system of an AGV vehicle. The proposed approach is based on the use of a 2D LiDAR sensor and the application of SLAM techniques using ICP, inspired by Hector SLAM, with the goal of enabling the robot to develop situational awareness of the environment and localize itself automatically, overcoming the limitations of the previous system based solely on a magnetic sensor.

To this end, SLAM techniques are explored in conjunction with variants of the ICP algorithm, evaluating their performance in real-world scenarios. In particular, the classical ICP, the Gauss–Newton-based ICP with point-to-point metric, and the one with point-to-plane metric are investigated, aiming to identify the approach that best balances accuracy and computational efficiency, considering the limitations of embedded hardware.

Based on these studies, an incremental mapping and localization system using 2D LiDAR was developed, capable of continuously estimating the robot’s pose and progressively building a map of the environment, characterizing a functional SLAM solution. This system was implemented to operate in real time on an embedded computing platform with processing constraints. Additionally, an interactive interface was developed for real-time visualization of the robot’s estimated position and the evolving map generated from LiDAR readings, using tools based on Qt.

Finally, the proposed solution was experimentally validated on a laboratory test platform AGV. The validation demonstrates the system’s potential to enhance the vehicle’s autonomy, enabling its reclassification from AGV to AMR and contributing to the adoption of more flexible and scalable solutions in industrial and logistics environments.

The proposed solution establishes a solid foundation for future trajectory planning and control strategies, allowing the AGV to operate more adaptively in structured environments.

The main contributions of this work are as follows:

- Integration of 2D LiDAR sensor: development of the necessary infrastructure for continuous acquisition and processing of scan data from a single sensor, without the need for complementary sensors;
- Comparative study of ICP variants: experimental evaluation of the performance of classical ICP and optimized variants using the Gauss–Newton method, with point-to-point and point-to-plane metrics, considering real data from the sensor to be integrated;
- Incremental execution of ICP: implementation of an architecture that performs continuous alignment between consecutive scans, enabling incremental pose estimation and progressive construction of the environment map;
- Embedded implementation and real-time graphical interface: adaptation of the system for operation on an embedded mini-PC with limited computational resources, along with the development of a Qt-based graphical interface for real-time visualization of the scans, estimated pose, and map evolution.

The article is organized into four main sections, including the introduction. Section 2 presents the materials and methods, covering the system design as well as the foundation and implementation of the ICP algorithm. Section 3 presents the results, including the evaluation of different ICP approaches, the simulation of the SLAM system using data captured from different positions, and the real-time validation with a 2D LiDAR on the AGV. Finally, Section 4 presents the study’s conclusions.

## 2. Materials and Methods

### 2.1. System Design

The methodology adopted in this work was structured to develop an efficient scan matching system based on the ICP method applied to a 2D LiDAR sensor. The work followed a sequence of steps: acquisition of data from the 2D LiDAR sensor; development of the ICP system to evaluate the efficiency of the proposed method; implementation of the incremental localization and mapping system; map smoothing and outlier removal; and finally, implementation of the real-time system and practical validation with the AGV vehicle.

**Figure 1:**
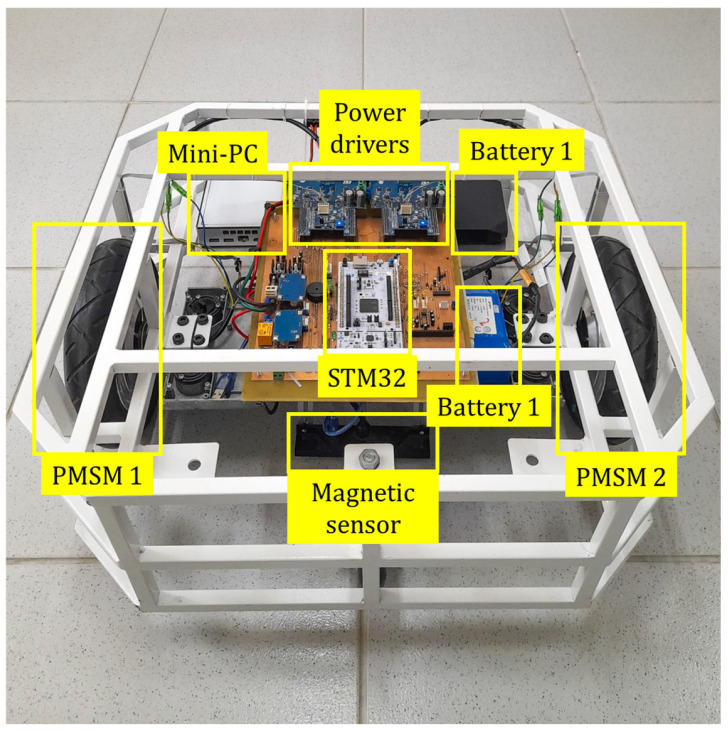
Main components of the AGV platform.

The vehicle in question is the robotic platform in the laboratory, composed of several essential components of the control and navigation system, as illustrated in Figure 1. The main components of the experimental platform are as follows: mini-PC with Intel^®^ Core™ i5-5200U processor and 8 GB RAM; STM32F767ZIT6U microcontroller; X-Nucleo-IHMO8M1 power drivers; MGS1600GY magnetic sensor; PMSM (Permanent Magnet Synchronous Motor) motors (350 W nominal power, powered at 36 V, with a peak current of 9.2 A); a battery bank with inverter (120 V, 60 Hz, 100 W output; dual 5 V 3 A USB ports; 20,400 mAh); and a 10S2P lithium-ion battery pack (36 V, 4400 mAh, 158.4 Wh). Based on the measured current drawn by both motors under typical load conditions (0.7 to 0.9 A each), the estimated power consumption is 65 W, resulting in an operational autonomy of 2 h 30 min.

**Figure 2:**
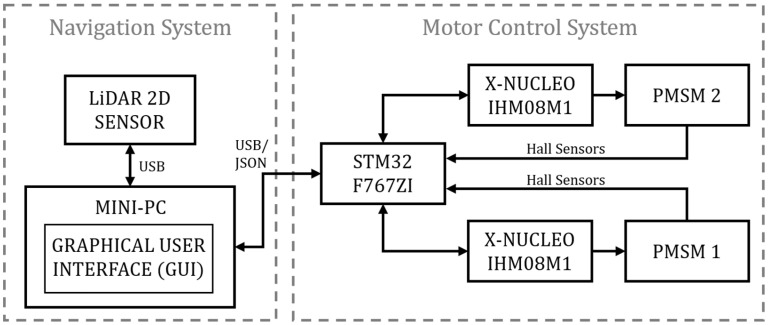
AGV platform systems.

It is possible to observe in Figure 2 the interconnection diagram of the components of the AGV proposed in this work. The mini-PC acts as the central processing unit, being responsible for managing and controlling the entire platform. The 2D LiDAR sensor operates as the main source of perception of the environment, being responsible for acquiring scans. It receives commands from the mini-PC to start or stop measurements and then transmits the scan data for processing. The control of the PMSM motors is carried out through the STM32 microcontroller, which receives commands from the mini-PC and is responsible for generating signals to the power modules (X-NUCLEO), actuating the motors. Additionally, the STM32 receives real-time feedback through the Hall effect sensors embedded in the motors. Communication between the mini-PC and the STM32 occurs via a USB connection using the JSON protocol, through which the STM32 receives the velocity parameters established by the navigation system.

**Figure 3:**
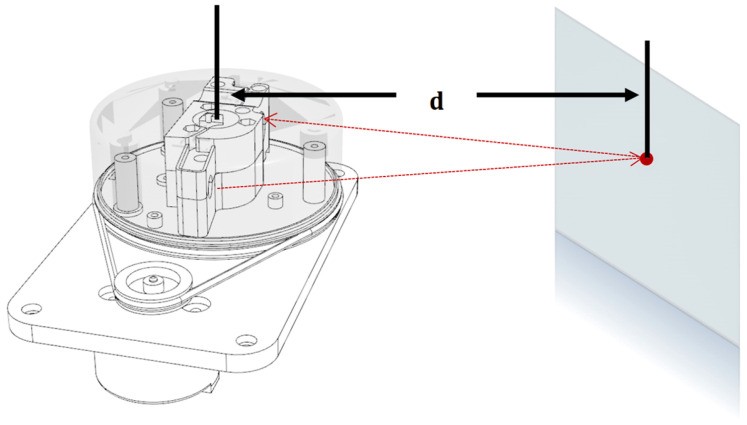
RPLIDAR A1 laser triangulation system.

The sensor used is the RPLIDAR A1M8 (Figure 3), a 2D LiDAR capable of performing 360° scans, widely used for mobile robot navigation to generate two-dimensional point clouds.

Its operation is based on laser triangulation measurement, in which a modulated infrared beam is emitted, reflected off objects in the environment, and detected by the sensor [21]. An integrated digital signal processor (DSP) calculates the distance d and the angle of each point from the return time of the light pulse. This time is obtained as the product of the number of counted pulses, n, and the interval τ, resulting in the expression

(1) $$d=\frac{1}{2}cn\tau ,$$

where d is the distance to the target, c is the speed of light, n is the number of clock pulses, and τ is the time interval between clock pulses. This technique is known as Time of Flight (ToF) measurement [22].

The RPLIDAR A1 features a rotary scanning module driven by a motor via belt, with continuous data reading via UART or USB. The scanning frequency automatically adjusts to the rotation, allowing the host system to monitor it in real time [3]. Its main advantages include low cost, a detection range of up to 12 m, a high sampling rate suitable for real-time applications, and a compact form factor. However, it also has limitations such as low angular resolution, sensitivity to intense sunlight, and restrictions when detecting reflective or transparent objects.

**Figure 4:**
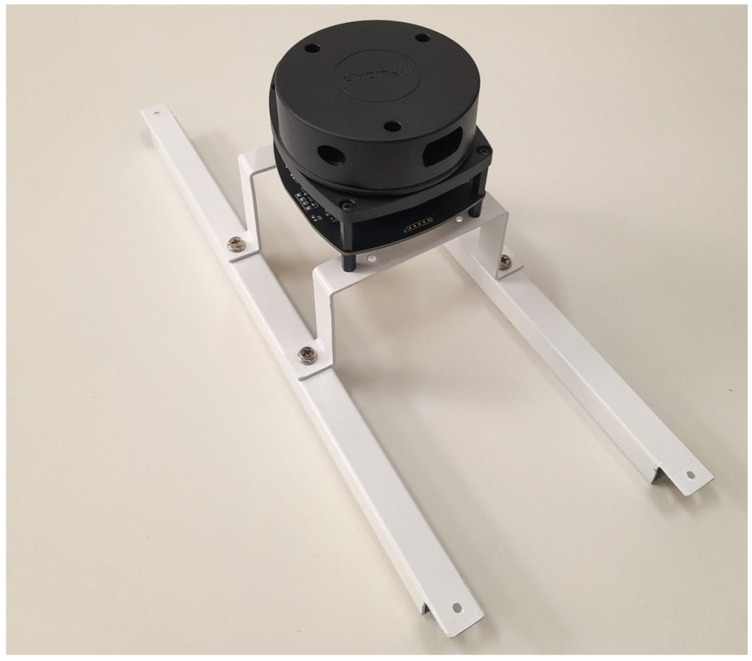
Metal structure with sensor to allocate to the platform.

To integrate the 2D LiDAR sensor into the vehicle, a metal structure was designed to be attached to the chassis, ensuring that the sensor remains centrally positioned. The final configuration of the support can be seen in Figure 4.

The proposed solution was implemented in C++ using Visual Studio 2022, with Eigen for linear algebra and Qt for graphical visualization. The choice of these tools ensures independence from external libraries such as ROS, greater portability, and lower computational cost compared to MATLAB version 24.2, which requires a license and has high processing demands.

### 2.2. ICP Algorithm and Implementation

The ICP algorithm is a technique for aligning 2D or 3D point clouds by estimating the rigid transformation that minimizes the distance between them [23,24]. The method is based on the correspondence between a point cloud and a reference cloud, typically obtained from 2D or 3D LiDAR sensors embedded in mobile robots [6]. Two point clouds in the Euclidean space $R^{d}$ are considered, defined as $P\;=\;{\left\{p_{i}\in R^{d}\right\}}_{i=1}^{N}$ and $Q\;={\left\{q_{j}\in \;R^{d}\right\}}_{j=1}^{M}$, where P is the fixed cloud and Q is the moving cloud to be aligned. The goal is to estimate a transformation matrix that minimizes the distance between corresponding points through an iterative process that reduces the sum of squared errors [6], given by

(2) $$E\left(R,t\right)=\arg\underset{R,t}{min}\sum_{i=1}^{N}∥p_{i}-\left(Rq_{j}+t\right){∥}^{2}.$$

To solve this minimization, it is common to use the Singular Value Decomposition (SVD) of the cross-covariance matrix between the centered point sets. SVD allows finding the optimal rotation that aligns the datasets through the expression

(3) $$R=UV^{⊺},$$

aligning the data with their principal directions [25]. The corresponding translation is obtained by

(4) $$t=\bar{p}-R\bar{q},$$

where $\bar{p}$ and $\bar{q}$ are the centroids of the sets P and Q, respectively. This procedure is repeated iteratively until convergence, forming the core of the classical ICP algorithm.

### 2.3. ICP Based on the Gauss–Newton Method

In practice, there are ICP variants that allow the algorithm to be optimized. In this case, it is necessary to move away from the SVD approach and adopt a general nonlinear least-squares formulation. The Gauss–Newton method, for instance, allows the incorporation of derivatives, which is key to obtaining an accurate iterative solution.

Considering Equation (2), the residual error for a pair of corresponding points is defined as

(5) $$e_{i,j}\left(x\right)=p_{i}-h_{j}\left(x\right)=p_{i}-\left(Rq_{j}+t\right),$$

where $x\;=\;{\left[x,\;y,\;\theta \right]}^{⊤}$ represents the pose parameters. To apply the Gauss–Newton method, the error function is linearized in the vicinity of the current estimate, resulting in the following linear system [26]:

(6) $$H\Delta x=-g⟹\Delta x=-H^{-1}g,$$

where Δx is the increment of the argument ([Δx,Δy,Δθ] in this case), H is the Hessian matrix of $e_{i,j}$, and g is the derivative of the error function $e_{i,j}$. The gradient g is computed as follows [26]:

(7) $$g=J^{⊤}e_{i,j}.$$

The elements of the Hessian are computed by differentiating the elements of the gradient [26], that is

(8) $$H=J^{⊤}J.$$

The computation of the gradient and the Hessian requires the Jacobian J. The Gauss–Newton method allows for different error metrics, with the most common in ICP being point-to-point, which minimizes the Euclidean distance between corresponding pairs, and point-to-plane, which projects the error in the direction normal to the surface, being more effective in planar regions.

For the point-to-point metric, the Jacobian of the error function with respect to the parameters [x, y, θ] is given by

(9) $$J_{\mathrm{point}\text{-}\mathrm{to}\text{-}\mathrm{point}}=\left[\begin{array}{ccc} 1 & 0 & -q_{x}sin\theta -q_{y}cos\theta \\ 0 & 1 & q_{x}cos\theta -q_{y}sin\theta \end{array}\right].$$

To use the point-to-plane metric, it is necessary to know the normal $n_{i}$ associated with the points in the fixed point cloud P. Each normal vector $n_{i}$ represents the orientation of the local plane around the point $p_{i}\in \;P$ and can be estimated by a least-squares fit to a small set of neighboring points [27]. Formally, the point-to-plane error to be minimized is expressed as follows [28]:

(10) $$E(R,t)=arg\;\underset{R,t}{min}\sum_{i=1}^{N}\|n_{i}\cdot (p_{i}-(Rq_{i}+t)){\|}^{2}.$$

Since the error is a scalar, the associated Jacobian takes the form of a 1× 3 matrix, given by

(11) $$J_{\mathrm{point}\text{-}\mathrm{to}\text{-}\mathrm{plane}}=\left[\begin{array}{ccc} n_{x} & n_{y} & n_{x}(-q_{x}sin\theta -q_{y}cos\theta )+n_{y}(q_{x}cos\theta -q_{y}sin\theta ) \end{array}\right].$$

In summary, while the point-to-point metric considers error in all directions, being suitable for general cases, the point-to-plane metric focuses on the most relevant component of the error, namely the surface normal direction. This makes it more accurate and efficient in structured environments. This choice reduces computational complexity, improves numerical stability, and accelerates algorithm convergence.

Based on the theoretical foundations discussed, the proposed approach is to use the Gauss–Newton method with the point-to-plane metric due to its greater efficiency in structured environments. For comparison purposes, two other variants were implemented: the classical ICP based on SVD and the Gauss–Newton ICP with a point-to-point metric, allowing the evaluation of the proposed method under the same conditions. Algorithm 1, developed in this work, presents the steps of the Gauss–Newton approach with the point-to-plane metric, reflecting the integration between mathematical concepts and the practical requirements for performance in mapping and localization systems.
**Algorithm 1:** ICP with Gauss–Newton Optimization
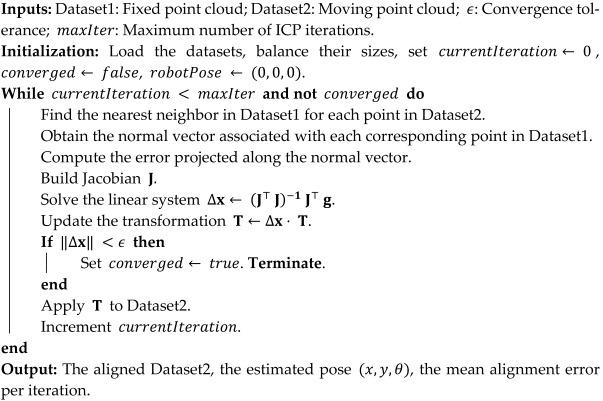


**Figure 5:**
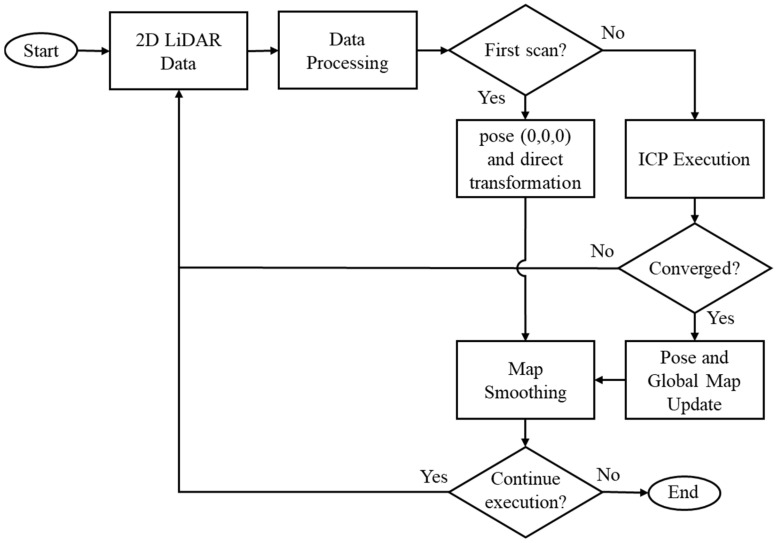
Simplified structure of the functional SLAM system.

In Figure 5, it is possible to observe the simplified flowchart of the 2D LiDAR-based mapping and localization system with ICP algorithm integration. The process begins with the acquisition and filtering of sensor data, eliminating low-reliability points. After acquisition, the data is filtered based on quality information provided by the sensor itself for each point, in order to discard unreliable measurements. The first scan is directly incorporated into the global map and used as a reference for future alignment.

Each new point cloud undergoes the same pre-processing. Next, the ICP algorithm is applied to align the mobile cloud (current) with the fixed (previous), estimating the transformation (displacement x, y, θ). This process is repeated until the convergence criteria are met. The robot pose is updated, and the points are transformed and incorporated into the global map. In the map smoothing stage, a filtering process is applied to remove redundant points and outliers, preserving only the most relevant information. The cycle repeats while there are scans to be processed or until the procedure is interrupted.

## 3. Results

### 3.1. Performance Evaluation of Different ICP Approaches

To evaluate the efficiency of the proposed method, the Gauss–Newton (GN) ICP with point-to-plane metric was compared to the classical point-to-point ICP and the Gauss–Newton optimized ICP with point-to-point metric.

**Figure 6:**
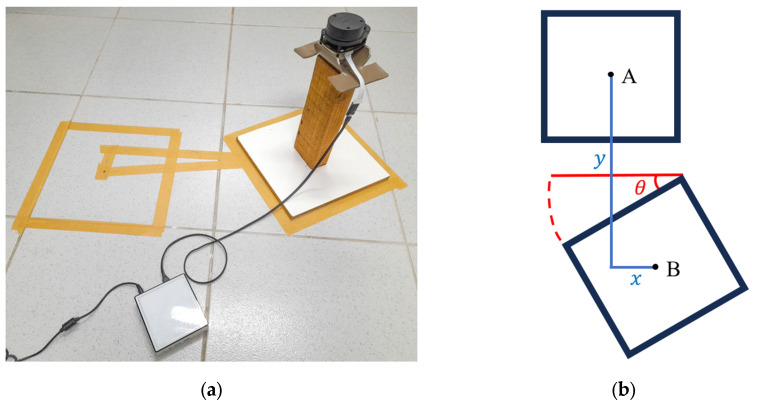
(**a**) Data collection with LiDAR for pose efference testing. (**b**) Schematic representation.

Data from a laboratory room were collected beforehand. The 2D LiDAR sensor was placed on a floor-mounted support (Figure 6a), simulating the height of a mobile robot. To ensure accuracy, floor markings defined two positions (A and B), allowing the measurement of displacements along the x and y axes and angular rotation, as illustrated in Figure 6b. In position A, the support was aligned with the tiles, while in position B it was rotated $30^{\circ }$, ensuring measurement consistency. The measured values were x=9.5 cm, y=55.5 cm, $30^{\circ }$. The data captured from both positions were stored in csv format for processing. This evaluation step was performed on a workstation equipped with an Intel^®^ Core™ i7-13700 processor and 16 GB of RAM.

**Figure 7:**
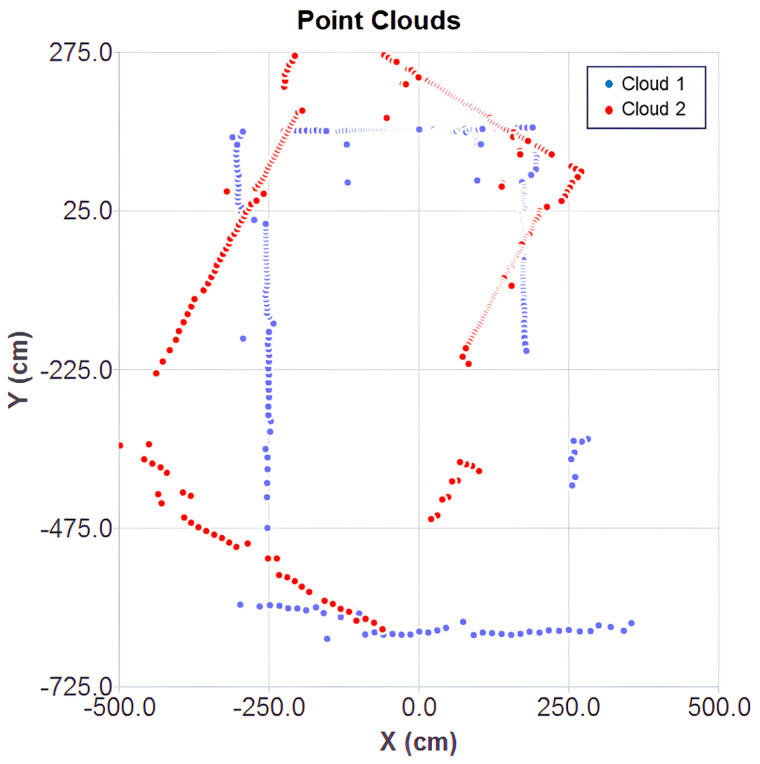
Origin position of point clouds.

The two point clouds are shown in Figure 7 under initial conditions, used as the starting point for each algorithm. Cloud 1 (in blue) serves as a reference for the alignment of cloud 2 (in red).

**Figure 8:**
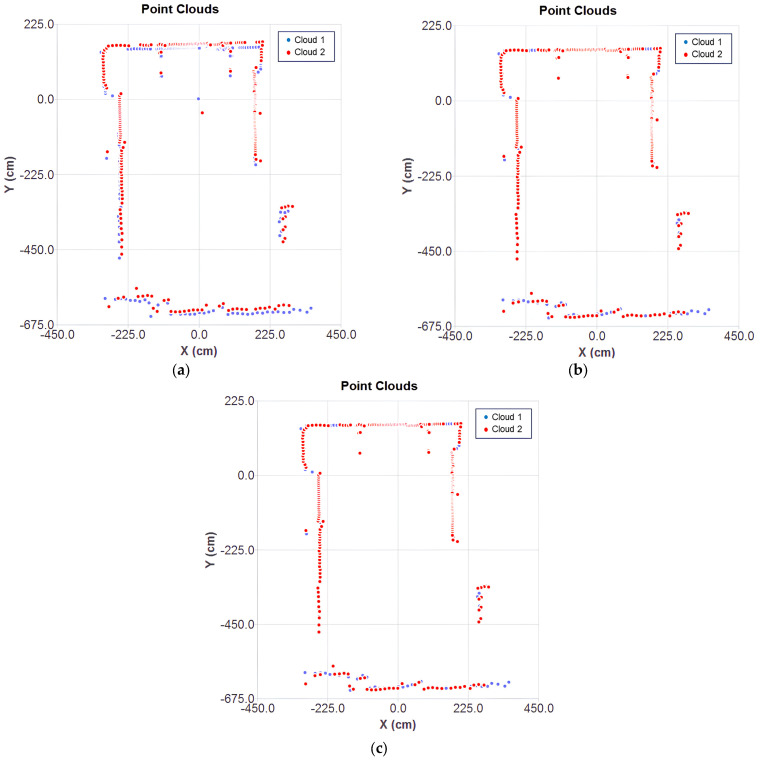
Converged cloud for each ICP variant: (**a**) classical method, (**b**) Gauss–Newton optimization with point-to-point metric, and (**c**) point-to-plane.

After convergence, the results of the aligned clouds for each ICP variant can be observed in Figure 8a–c. The classical point-to-point ICP showed noticeable misalignments, while the optimized cases ensured a better fit of the mobile cloud over the fixed one. The inaccuracy of the classical method stems from the direct point-to-point correspondence, without considering surface orientation. This effect is influenced by the mechanical deviations of the 2D LiDAR, making classical ICP more suitable for ideal data conditions. In contrast, the point-to-plane metric minimizes error based on the associative norm, resulting in more stable and consistent alignment to sensor noise.

**Figure 9:**
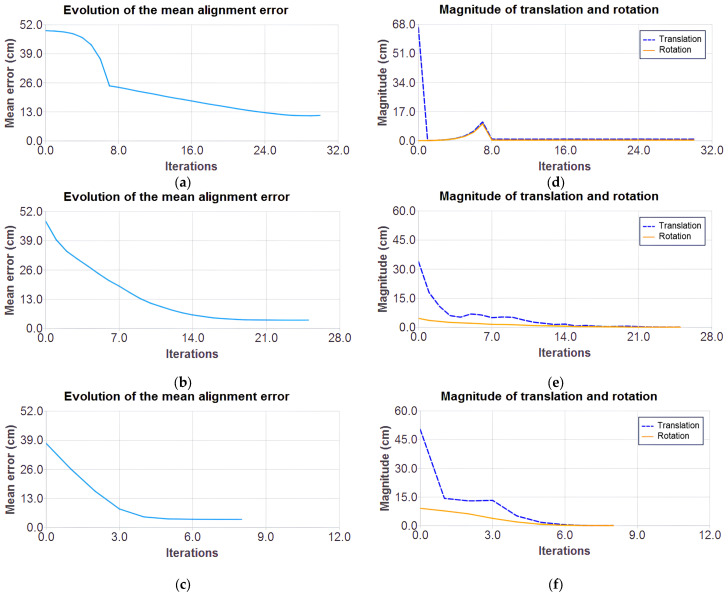
Mean error and the magnitude of translation and rotation over the iterations for each ICP variant: classical method (**a**,**d**), Gauss–Newton optimization with point-to-point metric (**b**,**e**), and point-to-plane (**c**,**f**).

Figure 9a–c show the error behavior over iterations, and Figure 9d–f show the magnitude of the cloud’s translation and rotation. In classical ICP, the error initially decreases rapidly and then stabilizes with the application of a movement threshold upon reaching a certain error for better refinement. It achieves a minimum error of 11.42 and converges at iteration 29, though with oscillations due to the exhaustive search mechanism.

In GN ICP with a point-to-point metric, the error reduction rate slows down, and the curve stabilizes, suggesting that the method has reached a stable minimum. Meanwhile, in the GN ICP method point-to-plane, the lowest error reaches 3.65 at iteration 7. This behavior reinforces its effectiveness in scenarios where the associative norms of the points are well-defined.

In the Gauss–Newton methods, convergence was determined by the minimum error variation (1 × 10^−8^), whereas in the classical method, the last valid transformation was retained if the error increased in the next iteration.

Table 2 summarizes the main results of the evaluated approaches, including estimated pose accuracy, average error, total execution time, and average CPU usage.

In terms of accuracy, the classic ICP presented the largest deviations between the estimated and real poses. The point-to-point GN ICP reduced the errors compared to the classic approach. The point-to-plane GN ICP achieved the highest accuracy, reaching 99.42% for x, 99.68% for y, and 99.99% for θ.

Regarding efficiency, the point-to-plane GN ICP consequently presented the lowest mean error (3.6528), followed by the point-to-point GN ICP (3.6972), while the classical ICP presented a significantly worse performance (11.4264). The classical ICP converged in 29 iterations, the point-to-point GN ICP in 23, and the point-to-plane GN ICP in only 7.

In terms of total execution time, which corresponds to the interval between the beginning and the end of the alignment process (including preprocessing, pose calculation, and update), the point-to-plane GN ICP took 278 ms, compared to 641 ms for the point-to-point GN ICP and 215 ms for the classic ICP. Despite requiring fewer iterations, the calculation of normals increased the total time of the point-to-plane method. Regarding the time spent per CPU, which refers only to the period of active processing by the CPU (excluding latencies and idle times), the classic ICP recorded the lowest value (6.88 ms), followed by the point-to-point GN-ICP (25.83 ms) and the point-to-plane GN ICP (27.56 ms). Finally, the average CPU utilization was 5.60% for classic ICP, 14.10% for point-to-point GN-ICP, and 13.62% for point-to-plane GN-ICP. Repeated testing showed minimal variations in these times, with no impact on the final accuracy, indicating deterministic behavior.

Therefore, the results confirm the superiority of the point-to-plane ICP with Gauss–Newton optimization since it presented the best performance, proving to be ideal for applications that require accuracy and fast convergence. These results formed the basis for the development of an incremental localization and mapping (SLAM) system, presented in the next subsection.

### 3.2. Offline SLAM: Processing Data Captured from Different Positions

**Figure 10:**
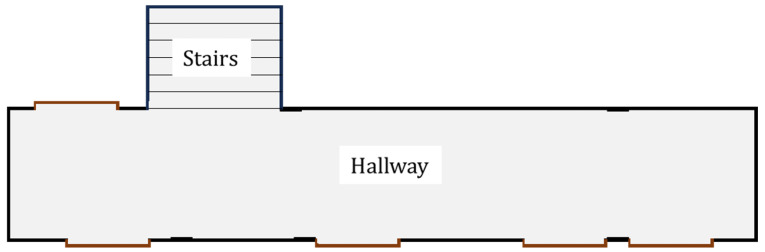
Format of the experiment environment.

The system was developed to process a database containing 16 point clouds (stored in csv files) captured by a 2D LiDAR sensor positioned at different locations along a laboratory hallway, simulating sequential readings in a scenario equivalent to the continuous operation of a mobile robot. This procedure enabled the creation of a controlled environment for system development, debugging, and validation before its real-time application with the AGV platform. The format of the area where the experiment was conducted is illustrated in Figure 10.

It is worth noting that, at this stage, no reference measurements were taken for the actual poses during each scan. Thus, the evaluation of the incremental ICP system was conducted qualitatively, based on the visual consistency of the estimated positions and the generated map, as well as prior knowledge of the environment’s geometry. Since the comparative study had already validated the accuracy of the adopted approach using real data, the focus of this stage was on the integration and incremental behavior of the system.

As illustrated in Figure 5, the adopted strategy is based on the alignment between consecutive scans. When executing the ICP processing, the resulting rigid transformation (x, y, θ) is used both to update the pose estimate and to reposition the moving point cloud relative to the global map.

After transforming the aligned points from ICP into the global coordinate system, a point redundancy filter was applied. The goal is to avoid inserting points that are too close to each other, preventing redundancy and excessive similar data in the map. Thus, the criterion was established: given a new point $p_{new}\;=(x_{new},\;y_{new})$, which is to be added to the global map, its Euclidean distance is checked in relation to all points already present in the set.

(12) $$d(p_{new},p_{i})=\sqrt{(x_{new}-x_{i}{)}^{2}+(y_{new}-y_{i}{)}^{2}}.$$

If there exists any point $p_{i}$ such that $d(p_{new},\;p_{i})\;<\;D\_min$, the point is discarded. In the code, this threshold is defined as $D_{min}=\;10\;cm$.

**Figure 11:**
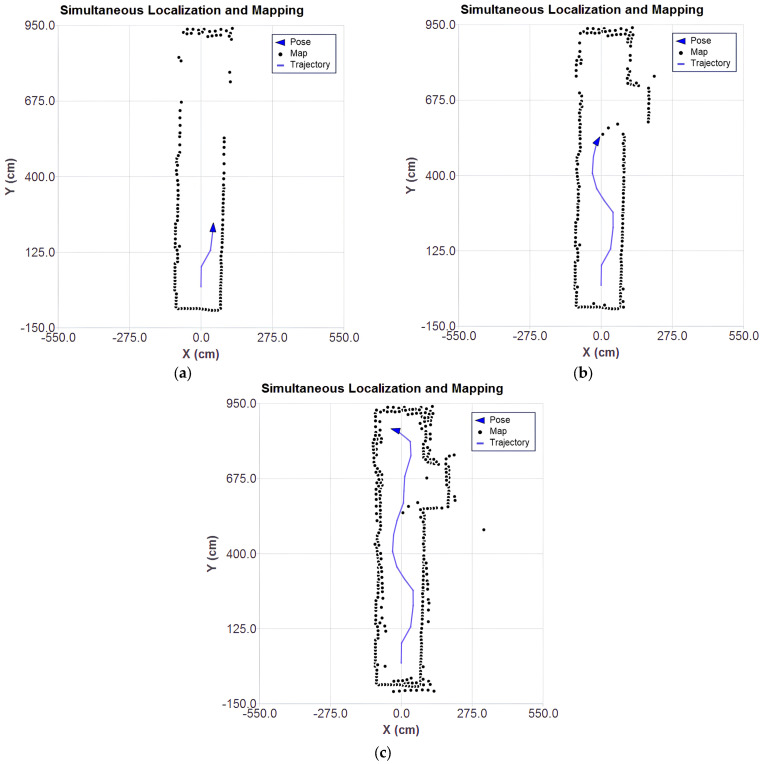
SLAM simulation with real data: (**a**) stage 1, (**b**) stage 2, (**c**) stage 3.

The results of the SLAM algorithm execution based on Gauss–Newton ICP with point-to-plane metric are presented at different stages in the plots of Figure 11a–c, which show the evolution of the mapping, the estimated position, and the trajectory throughout the processing of the captured scans.

As the LiDAR approaches the stairs (Figure 11b), there is a significant increase in the number of outliers. Although the system is filtering the data considered to be of higher quality by the sensor itself, it is still possible to observe inconsistent points. This inconsistency is associated with the limitations of the sensor. The base of the stairs is covered with a reflective material, which compromises the accuracy of the measurements.

In the final stage, shown in Figure 11c, after processing all the scans, the presence of scattered points can be observed, reinforcing the interference caused by the reflective material on the stairs. However, the robot’s pose estimation and trajectory remained consistent with the actual measurement positions.

To mitigate the limitations of the 2D LiDAR sensor, an outlier filter was implemented to improve the smoothing of the global map as new points are added. The goal is to identify and remove isolated points, that is, points with no nearby neighbors, since these are typically noise resulting from sensor errors. The defined criterion is as follows: for each point $p_{i}\;=\;(x_{i},\;y_{i})$, count how many neighbors $p_{j}$ (with j≠ i) are within a radius R. The count is performed as follows:

(13) $$\left(d\left(p_{i},p_{j}\right)=\sqrt{(x_{new}-x_{i}{)}^{2}+(y_{new}-y_{i}{)}^{2}}\right)<R.$$

If the number of neighbors of $p_{i}$ is less than a threshold $N_{min}$, then $p_{i}$ is considered an outlier and discarded from the global point cloud. In the code, R = 14 and $N_{min}=1$.

**Figure 12:**
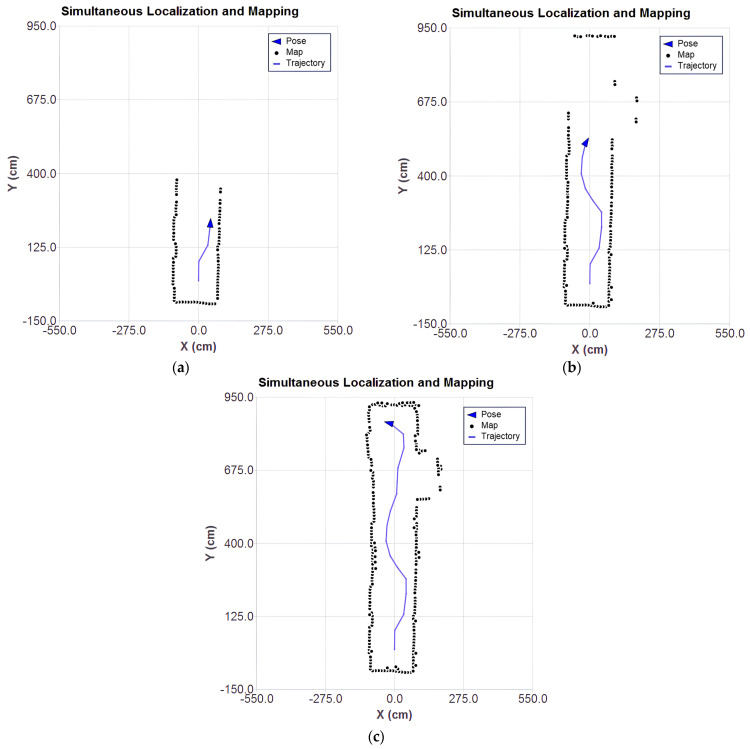
SLAM simulation with real data with outliers filtering: (**a**) stage 1, (**b**) stage 2, (**c**) stage 3.

Figure 12a–c present the results of the SLAM algorithm execution with the application of outlier filtering.

Compared to the previous results, it can be seen that the dispersion of spurious points was reduced, making the structure of the environment more clearly defined. This indicates that the filtering of redundant points and outliers was effective in removing noise and preserving only the most relevant points. The absence of points in the stair region is noticeable (Figure 12c). This absence is due to the reflective material present in that area, which compromises the reliability of the LiDAR sensor readings.

The final result suggests that the applied smoothing techniques contribute to more reliable mapping, reducing the effects of inaccurate LiDAR readings and ensuring that only trustworthy data are incorporated into the map. In the future, it is intended to explore other approaches to the removal of outliers discussed in the literature, such as those approached in [29].

### 3.3. Real-Time SLAM: Direct Implementation with 2D LiDAR

**Figure 13:**
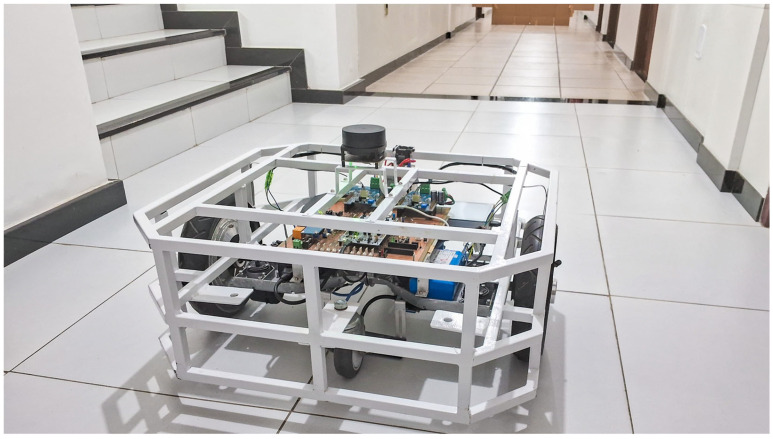
Visual record of the experiment with the AGV.

The objective of this experiment was to develop and test a real-time SLAM system, using data obtained directly from the LiDAR sensor integrated into the robotic platform to execute the proposed scan-matching algorithm. The platform with the integrated 2D LiDAR is shown in Figure 13 during a test in the experimental environment.

A graphical interface was developed in Qt/C++ for real-time visualization of the sensor scans, pose estimation, and map construction. The interface provides options to configure the LiDAR sensor’s communication parameters (baud rate and serial port), as well as dedicated buttons to start the LiDAR and the SLAM process. Additionally, the interface includes a specific section for configuring communication with the microcontroller responsible for motor control. In this section, it is possible to set the parameters (baud rate and serial port) and choose among three operating modes: Manual (direct motor start), Keyboard (motor control via keyboard keys), and 2D LiDAR (motor control using data obtained from the LiDAR).

**Figure 14:**
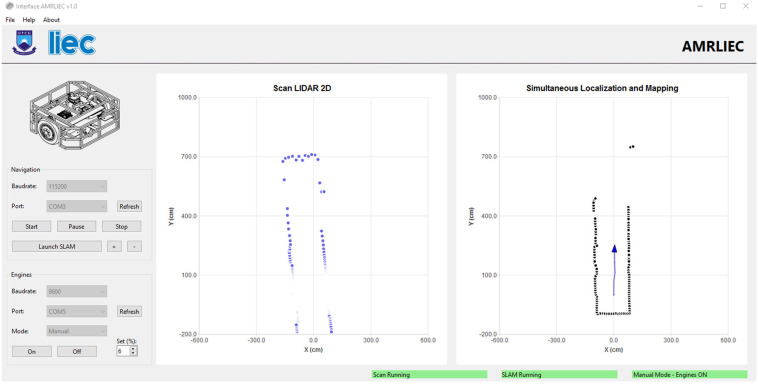
Graphical user interface running SLAM in real time (early stages).

**Figure 15:**
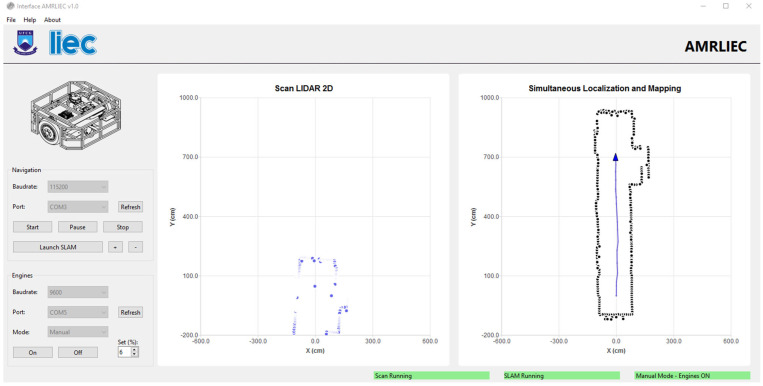
Graphical user interface running SLAM in real time (final stages).

The initial map construction is presented in Figure 14, where the general structure of the environment can already be identified. In Figure 15, as the robot moves, the progression of the mapping is shown with the successive incorporation of new scans into the global map.

Due to the hardware limitations of the embedded mini-PC, the localization and map updates occur every 3 s. The system uses asynchronous threads to better utilize the processing cores. However, when testing an update rate of 1 s, the interface became unresponsive, as the class responsible for ICP was still running while new LiDAR data continued to arrive. The 3-s rate was considered ideal, as ICP takes between 2 and 2.5 s to converge, allowing for pose updates and the addition of new points to the map without compromising system stability.

One of the main challenges faced during the experiments was the limitation of the motor control system on the robotic platform. The lack of torque control made it difficult to perform low-speed movements, which directly impacted the ability to operate the robot through manual commands or automatically using LiDAR data. This limitation affected the quality of some experiments, since SLAM systems tend to perform better at lower speeds and with more controlled movements. The vehicle’s wheels have a diameter of 26 cm, and during the real-time experiment, the motors were operated at approximately 10 rpm, which ensured smooth movement and consistency in the environment mapping. This rotational speed corresponds to a linear velocity of 0.136 m/s.

The proposed real-time functional SLAM system demonstrated consistency with the objectives, but its performance may vary depending on the environment. In places with thin obstacles, such as tables and chairs, the map representation may be compromised, as well as the pose estimation. This is because in the LiDAR scan, these objects generate sparse or distorted points, making precise alignment in the point-to-plane ICP algorithm more difficult. It is worth noting that this type of vehicle is recommended for operation in structured and flat environments. Additionally, the robot’s movement speed also influences performance; smoother displacements result in more consistent localization and mapping.

## 4. Conclusions

This work presented the development of a real-time incremental mapping and localization system, focusing on enhancing the perception of an AGV previously limited to the use of a magnetic sensor for navigation. The proposal responds to demands identified in the literature for efficient SLAM solutions for embedded platforms with limited computational resources, eliminating the need for auxiliary sensors or previous maps. Additionally, the system adopts a modular architecture and supports real-time visualization and validation through a graphical interface compatible with Windows and Linux, overcoming limitations of conventional approaches restricted to the Linux environment.

All proposed objectives were achieved: the integration of a 2D LiDAR sensor into the AGV, the comparative evaluation of ICP algorithm variants, the implementation of an incremental SLAM system, and the development of a graphical interface for real-time monitoring.

The experiments demonstrated that the ICP-based approach with Gauss–Newton optimization and point-to-plane metric offers high accuracy in pose estimation within a controlled scenario. In both simulated and embedded tests of the developed system, even in the absence of true position measurements at the time of capture of each scan, qualitative evaluation indicated coherence in the estimates and visual plausibility with the data acquisition environment. The filtering applied to the global map contributed to a cleaner and more reliable representation, reducing the effects of sensor noise. Finally, the embedded operation was successful, maintaining performance compatible with mobile robotics applications, even with a moderate update rate. These results reinforce the feasibility of the proposal for practical applications in structured environments.

Furthermore, the developed modules provide a solid foundation for future system extensions, including trajectory planning and control strategies, increasing the AGV’s degree of autonomy. The proposed solution shows potential for industrial, academic, and educational applications, contributing to the advancement of more autonomous, precise, and adaptable perceptual systems.

The following directions for future investigations are highlighted: (i) quantitative evaluation of the system’s accuracy through real-time pose measurements during operation; (ii) sensor fusion with odometry or camera data to enhance robustness; and (iii) development of complementary modules for navigation planning and control based on the generated map, expanding the platform’s autonomy and practical applicability.

## Figures and Tables

**Table 1:** Comparative overview of SLAM approaches.

Approach	Sensor Type	Odometry Dependency	Main Method	Computational Complexity	Applications	Features
Hector SLAM [14].	2D LiDAR.	No.	Direct Scan Matching.	Medium.	Mobile robots, drones.	High-frequency; lacks loop closure.
Gmapping [9].	2D LiDAR + Odometry.	Yes.	Particle Filter.	High.	Mobile robotics.	Robust, but sensitive to odometry errors.
ORB-SLAM [8].	Camera (Monocular, RGB-D).	No.	Visual Features (ORB).	Medium.	Augmented Reality, robotics.	Requires rich visual features; loop closure supported.
LOAM [11,12].	3D LiDAR + IMU.	Yes.	Geometric Feature Extraction.	High.	Drones, autonomous vehicles.	High accuracy; computationally demanding.
Proposed work.	2D LiDAR.	No.	Optimized ICP (Gauss-Newton).	Medium.	Mobile robotics.	Balanced accuracy and computational cost; modular architecture.

**Table 2:** Performance results of each approach.

Evaluation	ICP (Classical)	ICP-GN (Point-to-Point)	ICP-GN (Point-to-Plane)
True pose (x, y, θ) (cm)	9.5; −55.5; 30	9.5; −55.5; 30	9.5; −55.5; 30
Est. pose (x, y, θ) (cm)	10.8063; −42.0348; 30.5742	9.4059; −54.9814; 30.0578	9.5554; −55.3249; 30.0014
Accuracy (x, y, θ) (%)	86.25; 75.75; 98.09	99.01; 99.07; 99.81	99.42; 99.68; 99.99
Average error (cm)	11.4264	3.6972	3.6528
Iterations	29	23	7
Total execution time (ms)	215	641	278
Time spent by CPU (ms)	6.8834	25.8368	27.5617
Avg. CPU utilization (%)	5.6029	14.1001	13.6279

## Data Availability

The data presented in this study are available on request from the corresponding author.
